# Hippocampal Volume Is Smaller In Female Double Carriers Of Two Strongest AD Genetic Risk Factors

**DOI:** 10.1093/geroni/igab046.2461

**Published:** 2021-12-17

**Authors:** Svetlana Ukraintseva, Vladimir Popov, Konstantin Arbeev, Hongzhe Duan, Olivia Bagley, Alexander Kulminski, Anatoliy Yashin

**Affiliations:** 1 Duke University, Durham, North Carolina, United States; 2 DUKE UNIVERSITY, Durham, North Carolina, United States

## Abstract

Genetic risk factors for Alzheimer’s disease (AD) may facilitate AD-related changes in the brain long before AD clinical manifestation. While APOE4 was linked to a reduced hippocampal volume (HV) in a number of studies, the impact of rs2075650, another polymorphism strongly associated with AD, on HV is less clear. The rs2075650 (in TOMM40) is only in moderate to low LD with APOE4, and may have independent effects on HV or interact with APOE4. We studied associations of rs2075650 (G allele, risk factor for AD), rs429358 (C allele, proxy for APOE4), and their combinations, with right HV measured by MRI, among 10,738 women and 9,775 men aged 60-75, from UK Biobank. We found that right HV was significantly (p<0.02) smaller in women who carry both AD risk variants (rs2075650(G) and rs429358(C)), than in non-carriers of both of these variants, while having only one risk variant (G or C) didn’t clearly affect HV. The studied associations didn’t reach statistical significance in men. Our results suggest that rs2075650(G) and rs429358(C) may contribute synergistically to a reduction in hippocampus volume, in females only, and support the role of interactions between genetic risk factors for AD in sex differences in preclinical biomarkers of AD pathology.

